# Sex-dependent alterations of colonic epithelial permeability: relevance to irritable bowel syndrome

**DOI:** 10.3389/fphys.2025.1509935

**Published:** 2025-02-21

**Authors:** Muriel Larauche, Swapna Mahurkar-Joshi, Mandy Biraud, Tiffany Ju, Emeran A. Mayer, Lin Chang

**Affiliations:** ^1^ Vatche and Tamar Manoukian Division of Digestive Diseases, David Geffen School of Medicine, University of California, Los Angeles, Los Angeles, CA, United States; ^2^ VA Greater Los Angeles Healthcare System, Los Angeles, CA, United States

**Keywords:** IBS, permeability, colon, biopsies, sex differences

## Abstract

**Introduction:**

Increased intestinal permeability is a possible pathophysiological mechanism of irritable bowel syndrome (IBS). Increased colonic epithelial permeability is associated with visceral nociception in rodents and abdominal pain severity in IBS patients. Although IBS is more common in women, most studies on IBS-associated epithelial dysfunction have largely overlooked sex as a biologic variable.

**Methods:**

Men and women with Rome III- and Rome IV-positive IBS and HCs rated GI symptoms including abdominal pain severity at baseline, 24 h prior and immediately post procedure. Epithelial function was assessed *ex vivo* in Ussing chambers using sigmoid colon biopsies, by monitoring short-circuit current (Isc), transepithelial electrical resistance (TEER) and mucosal permeability to FITC-dextran 4 kDa (FD4). Biopsies tight junction protein mRNA expression was assessed using RNA seq. Statistical analyses included a framework of General Linear Models and linear contrast analyses performed using R software.

**Results:**

44 IBS patients (66% women, 30 years) and 19 HCs (53% women, 28 years) were enrolled. The proportion of women was not different between groups. As a group, IBS patients exhibited lower TEER compared to HCs (16.9 ± 5.5 vs. 21.5 ± 6.5 Ω/cm^2^, p = 0.01, FDR = 0.02), but no difference in FD4 serosal concentration or Isc (basal or stimulated). Within men, IBS had lower TEER vs. HCs, but there was no disease difference within women. Independent of diagnosis, women had 1.3-fold lower TEER concentration and 1.5-fold higher FD4 serosal concentration than men. These sex differences were also seen within HCs, although within IBS, FD4 permeability only showed a trend to be higher in women vs. men. Abdominal pain ratings and IBS severity scores were not associated with TEER or FD4 concentration.

**Discussion:**

Our study confirms prior reports that IBS patients demonstrate altered sigmoid colonic epithelial function and shows for the first time that these are independent of sex. However, sex differences in sigmoid colonic epithelial function are observed independently of disease status. Further studies are needed to delineate if intestinal permeability interacts with other factors in the pathophysiology of IBS and if these interactions differ by sex.

## 1 Introduction

Irritable bowel syndrome (IBS) is one of the most common disorders of gut-brain interactions (DGBI), and depending on the applied diagnostic Rome criteria, has a prevalence of 10.1% (Rome III) and 4.1% (Rome IV) worldwide (Sperber et al., 2021). IBS patients present with abdominal pain and variable alterations in bowel habits, which define the subtype of IBS. These subtypes are diarrhea-predominant IBS (IBS-D), constipation-predominant IBS (IBS-C), mixed IBS (IBS-M) or unspecified IBS (IBS-U) (Enck et al., 2016). Abdominal pain is the most important determinant of IBS severity, quality of life impairment and healthcare utilization (Spiegel et al., 2010). Visceral hypersensitivity to rectosigmoid distension is an important hallmark feature of IBS, believed to underlie abdominal pain in patients. It is estimated that 47%–64% of IBS patients have lower rectal discomfort thresholds compared to HCs (Chang et al., 2006). IBS is sexually dimorphic in terms of symptoms, and responsiveness to drugs, and predominantly affects women (about 2:1), but the underlying mechanisms of these sex related differences are incompletely understood (Black and Ford, 2020).

A compromised epithelial barrier function has been demonstrated in subsets of IBS patients with either IBS-D or IBS-C (Hanning et al., 2021) and more recently IBS-M symptoms (Awad et al., 2023). The prevalence of increased permeability in IBS patients is variable (2%–62%), with inconsistent findings (JohnBritto et al., 2024), and different underlying structural alterations (Bertiaux-Vandaële et al., 2011) across subtypes. While elevated permeability ranging from 37% to 62% is described in IBS-D patients (Hanning et al., 2021), and consistently reported in post-infection IBS (PI-IBS) patients (Spiller et al., 2000; Dunlop et al., 2006), reports in IBS-C are mixed (Hanning et al., 2021). Perturbations in epithelial barrier function using functional or structural assays have been described throughout the digestive tract of IBS patients (Barbara et al., 2024), and noted in the duodenum, jejunum, ileum, cecum, ascending colon, descending colon, and rectosigmoid colon (JohnBritto et al., 2024). Importantly, increased colonic epithelial permeability is associated with abdominal pain severity in IBS patients (Hanning et al., 2021; Zhou et al., 2009; Camilleri et al., 2012; Witt et al., 2019) and visceral hypersensitivity in rodents (Ait-Belgnaoui et al., 2005; Creekmore et al., 2018), making it an important pathophysiological mechanism underlying pain in IBS patients (Hanning et al., 2021).

Interestingly, although IBS is more common in women, most studies on IBS-associated epithelial dysfunction have largely overlooked sex as a biologic variable (SABV). There is evidence, although not always consistent, both in humans (Edogawa et al., 2018; Maget et al., 2021) and in rodents (Volynets et al., 2016) that sex may affect intestinal permeability and that sex hormones potentially play a role in this modulation (Flood et al., 2022; Lambert et al., 2012; Shieh et al., 2020). Sex hormones may directly affect tight junction (TJ) expression (Braniste et al., 2009; Zhou et al., 2017; Looijer-van Langen et al., 2011) or target mast cells (Mackey et al., 2016) and the immune system (Homma et al., 2005) which are well known contributors to epithelial barrier alterations (Albert-Bayo et al., 2019; Shea-Donohue and Urban, 2017).

In this study, we therefore aimed to determine if sigmoid colonic epithelial function differs: 1) between IBS and healthy controls (HCs), 2) by sex, and 3) by bowel habit predominance. Additionally, we investigated baseline and cholinergic muscarinic-evoked mucosal secretory properties and colonic TJ gene expression in men and women with IBS-C and IBS-D in comparison to HCs.

## 2 Materials and methods

### 2.1 Study subjects and recruitment

Male and female participants aged 18–55 years who fulfilled Rome III and Rome IV diagnostic criteria for IBS (Longstreth et al., 2006; Drossman, 2016) recruited from community were included in this study. Subjects underwent a medical history and physical examination by a gastroenterologist with expertise in IBS (LC). At the screening visit, a history and physical examination and structured psychiatric interview (Mini International Neuropsychiatric Interview [MINI]) were performed (Sheehan et al., 1998). The diagnosis of IBS was determined based on the Rome criteria (Munakata et al., 1997). HCs had no personal or family history of IBS or other chronic pain disorders. Additional exclusion criteria for all subjects included: pregnancy, infectious or inflammatory disorders, active psychiatric illness over the past 6 months as assessed for the DSM-IV (MINI), use of corticosteroids in the past 6 months, use of narcotics, antidepressants, or current tobacco or alcohol abuse. Subjects were compensated for participating in the study. Informed consent was obtained from all subjects. The study was approved by the UCLA Institutional Review Board and was conducted in accordance with the institutional guidelines regulating human subjects research. All subjects gave their written informed consent.

### 2.2 Symptoms measure

At the screening visit, a bowel symptom questionnaire was used to assess the presence and severity of IBS symptoms and duration of disease (Munakata et al., 1997). It included the Rome III and Rome IV diagnostic questions for IBS, bowel habit subtypes, demographic characteristics, current abdominal pain severity (0–20), and usual IBS severity [“How bad are your symptoms usually?” None (0) to very severe (5)]. IBS symptom severity was also measured using a validated measure of IBS symptom severity, IBS Severity Scoring System (IBS-SSS) (Francis et al., 1997), which assesses severity of abdominal pain, frequency of abdominal pain, severity of abdominal distention, dissatisfaction with bowel habits, and interference of IBS with daily life over a 10-day period. Each of the five categories received a score from 0 to 100, and the total IBS-SSS was calculated by taking the sum of these categories (total score range 0–500; >75: remission <75, mild 75–175, moderate 175–300, severe >300). Patients were asked to rate their discomfort and intensity of abdominal pain in the 24 h prior and immediately post biopsy collection.

Validated questionnaires were administered to patients and HCs to assess psychological symptoms. The Hospital Anxiety and Depression Scale (HAD) is a widely used 14-item questionnaire for assessing current symptoms of anxiety and depression (Zigmond and Snaith, 1983).

### 2.3 Sigmoid colon biopsy collection

A flexible sigmoidoscopy to at least 30 cm from the anal verge was performed. Subjects were instructed to use two tap-water enemas as the bowel preparation. During the sigmoidoscopy, 20 sigmoid colon biopsies were taken at 30–40 cm from the anal verge. The first 4 biopsies were used for permeability assays in Ussing chambers, the others were used for mRNA expression assay and banking. For premenopausal women not taking oral contraceptive agents, menstrual cycle phase was determined by the count forward/backward method (menses: first 3 days of menses; follicular: days 4–14; luteal: day 14 to onset of menses). Serum progesterone was collected to help confirm cycle phase.

### 2.4 Electrophysiological and permeability measurements in biopsies

Immediately upon collection, biopsies were placed in oxygenated (carbogen) Krebs-Ringer solution containing (in mM): 115 NaCl, 1.2 CaCl2, 1.2 MgCl2, 4.8 KH2PO4, 48 K2HPO4, 25 NaHCO3, and 10 glucose. The solution was bubbled with 95% O_2_-5% CO_2_ to maintain pH at 7.4 and kept on ice for transport to the laboratory. Less than 30 min post collection, biopsies were mounted in 0.031 cm^2^ sliders dedicated for Ussing chambers (Physiologic Instruments, San Diego, CA, United States) and bathed with Krebs-Ringer solution kept at 37°C during the course of the experiments by a circulating water bath heater.

Tissues were left to equilibrate in the chambers for 30–45 min before conducting the experiments. The tissues were short-circuited by a voltage clamp (VCC MC6; Physiologic Instruments, San Diego, CA, United States) at zero potential automatically with compensation for solution resistance. The short-circuit current (Isc), a measure of basal rheogenic anion secretion, and transepithelial electric resistance (TEER) were determined every 2 s and recorded by the DataQ system (Physiologic Instruments, San Diego, CA, United States). Positive values for Isc indicate a negative electrical charge flux from serosal → luminal bath indicating anion secretion or cation absorption. Epithelial permeability was monitored by measuring the mucosal-to-serosal transepithelial passage of fluorescein-isothiocyanate (FITC) dextran 4 kDa (FD4; Sigma-Aldrich, St Louis, LA, United States). Samples from the serosal chamber were collected every 30 min for 2 h. At the end of the experiment, the muscarinic agonist carbachol (CCh, 10 μM, Sigma-Aldrich, St Louis, LA, United States) was administered on the serosal side to assess the maximal secretory capacity of the epithelium, in order to test the viability of the tissue under *in vitro* conditions and the secretory response to a muscarinic stimulation. Intestinal permeability to FD4 was determined by measuring FD4 concentration in the samples using an automatic Synergy HT multi-detection microplate reader (Ex 485 nm; Em 525 nm, BioTek, Winooski, VT, United States). The mucosal-to-serosal flux of FD4 was determined and expressed in ng/h/cm^2^. The final 2 h cumulative serosal concentration of FD4 (ng/mL) and the slope of the FD4 flux (a.u.), calculated by plotting the flux as a function of time of collection (0, 30, 60, 90 and 120 min) and applying a linear regression, were also determined.

### 2.5 RNA seq: tight junction protein gene expression

Gene expression was measured using QuantSeq 3′ RNA sequencing (3′RNA-Seq). 3′RNA-Seq data processing was performed using Methods described previously (Mahurkar-Joshi et al., 2021). Briefly, Bluebee^©^ Genomics platform (https://www.bluebee.com/lexogen/) was used to demultiplex reads. This pipeline includes trimming of adapter and polyA sequences and low-quality nucleotides using BBDuk (http://jgi.doe.gov/data-and-tools/bb-tools/), alignment of trimmed reads against the human genome (GRCh38) with STAR^35^, and determination of gene counts with HTseq^36^. We evaluated the expression of 25 genes including, claudins (CLDN) 3, 4, 5, 7, 8, 12, 15, 23; occludin (OCLN); ZO-1 (TJP-1); ZO-2 (TJP-2); ZO-3 (TJP-3), cingulin (CGN), paracingulin or cingulin-like-1 (CGNL1), JAM-A (F11R), JAM-B (JAM2), JAM-C (JAM3), pleckstrin homology domain containing A7 (PLEKHA7), myosin light chain kinase, MYLK. We also assessed the expression of the cholinergic muscarinic receptor 3 (CHRM3) and mast cell proteases carboxypeptidase A3 (CPA3) and tryptase alpha/beta 1 (TPSAB1).

### 2.6 Salivary sex hormones levels

Prior to the collection of biopsies, patients were asked to provide salivary samples while lying supine. The saliva was then transferred into a cryotube by pouring it in directly, flash frozen in liquid nitrogen and then stored at −80°C until processing. Salivary 17β-estradiol (kit# 1-3702) and progesterone (kit# 1-2502) levels in IBS and HC women were assayed using EIA kits according to the manufacturer’s instructions (Salimetrics LLC, PA, United States).

### 2.7 Statistical analysis

#### 2.7.1 Permeability data analysis

Group differences in demographic and permeability measures (Isc, TEER, CCh, FD4, and FD4 slope) between the diagnostic groups (IBS vs. HC) and sexes (women vs. men) were analyzed using general linear model (GLM). Models assessing group differences between IBS vs. HC included sex as a covariate. We used GLMs with linear contrast analyses (LCA) to test differences between IBS × sex interaction groups (female IBS vs. male IBS, female HC vs. male HC, female IBS vs. female HC, male IBS vs. male HC, female IBS - male IBS vs. female HC - male HC). Permeability data were log_2_ transformed after the removal of outliers before running the GLMs. Linear regression with sex as a covariate was used to analyze relationships between permeability measures, genes and clinical traits in IBS patients. An FDR of <5% as significant, correcting for the six permeability measures tested. To test IBS diagnostic group differences, sex differences, and IBS diagnostic x sex group differences between baseline and post CCh treatment, we used linear mixed-effects models. The p-values were adjusted for multiple comparisons for the number of variables tested using FDR.

#### 2.7.2 3′RNA-seq data analysis

Differentially expressed genes between IBS and HC groups and sexes were analyzed using GLMs including sex and sequencing batch as covariates for IBS vs. HC models and sequencing batch as a covariate for testing group differences between female vs. male participants. GLM with LCA was used to test differences in gene expression between IBS × sex interaction groups (female IBS vs. male IBS, female HC vs. male HC, female IBS vs. female HC, male IBS vs. male HC, female IBS–male IBS vs. female HC–male HC). Data were transformed using centered log-ratio (CLR) transformation before performing the GLM LCAden (Van den Boogaart KGT-D, 2013). The sequencing batch was used as a covariate in all the models and sex was used as a covariate for IBS vs. HC comparison. Linear regression with sex as a covariate was used to analyze relationships between gene expression, IBS symptom severity and permeability measures. An FDR of <5% as significant, correcting for the 25 genes tested. All the statistical analyses and visualization were performed using R statistical analysis software (http://cran.r-project.org/).

Data are presented as mean ± SD, graphical representation as mean ± SEM.

## 3 Results

### 3.1 Clinical characteristics of study participants

Forty-four IBS patients (29 women and 15 men) and 19 HCs (10 women and 9 men) participated in the study. The clinical, behavioral and demographic data are shown in Table 1. Mean age, BMI, sex distribution (53%–66% women), and race/ethnicity of the IBS and control groups were similar (Table 1). Based on predominant bowel habit, IBS subjects were classified into the following subtypes: IBS-C = 8 women/3 men, IBS-D = 9 women/7 men, IBS-M = 9 women/2 men and IBS-U = 3 women/3 men. IBS patients reported significantly higher scores for HAD anxiety and depression symptoms (all p’s < 0.05). The mean (SEM) IBS-SSS was 207.65, indicating moderate severity of IBS symptoms in this cohort of patients.

**TABLE 1:** Characteristics of study population.

	IBS (n = 44) (mean ± SD)	HC (n = 19) (mean ± SD)	t or chi-squared value	p-value
Age	29.98 (10.68)	28.11 (10.26)	0.65	0.52
Sex (% women)	29 (66)	10 (53)	0.51	0.399
BMI	24.99 (3.94)	26.17 (4.03)	1.09	0.28
Bowel habit subtypes				
IBS-C (n)	11 (8F/3M)	NA	NA	NA
IBS-D (n)	16 (9F/7M)	NA	NA	NA
IBS-M (n)	11 (9F/2M)	NA	NA	NA
IBS-U (n)	6 (3F/3M)	NA	NA	NA
Anxiety	7.70 (3.56)	2.68 (3.00)	5.35	1.5e-06*
Depression	3.58 (3.27)	1.11 (1.76)	3.10	3e-03*
Overall Severity (0–20)	8.68 (4.12)	NA	NA	NA
Abdominal Pain (0–20)	8.23 (3.54)	NA	NA	NA
Bloating (0–20)	9.41 (4.14)	NA	NA	NA
Intensity of abdominal symptoms at 24 h (0–20)	8.6 (4.26)	NA	NA	NA
Unpleasantness of abdominal symptoms at 24 h (0–20)	7.8 (3.89)	NA	NA	NA
Intensity of abdominal symptoms immediately after the procedure (0–20)	10.88 (4.04)	NA	NA	NA
Unpleasantness of abdominal symptoms immediately after the procedure (0–20)	9.15 (3.77)	NA	NA	NA
IBS-SSS (0–500)	207.65	NA	NA	NA

Bold values indicate statistically significant differences.

Amongst women, 10 were in follicular phase (7 IBS and 3 HCs) and 18 in luteal phase (14 IBS and 4 HCs). The rest were either taking control-birth medications [3 IUD (2 HCs, 1 IBS)], 1 progestin implant, or menstruating (1 at day 2) and were considered as unknown phase; or were considered other (2 hysterectomy, 2 post-menopausal, 1 etonogestrel implant; all IBS).

### 3.2 Epithelial permeability: TEER and FD4

#### 3.2.1 Differences in permeability measurements between IBS and HCs, sexes and IBS × sex interaction groups

##### 3.2.1.1 IBS vs. HCs

**FIGURE 1:**
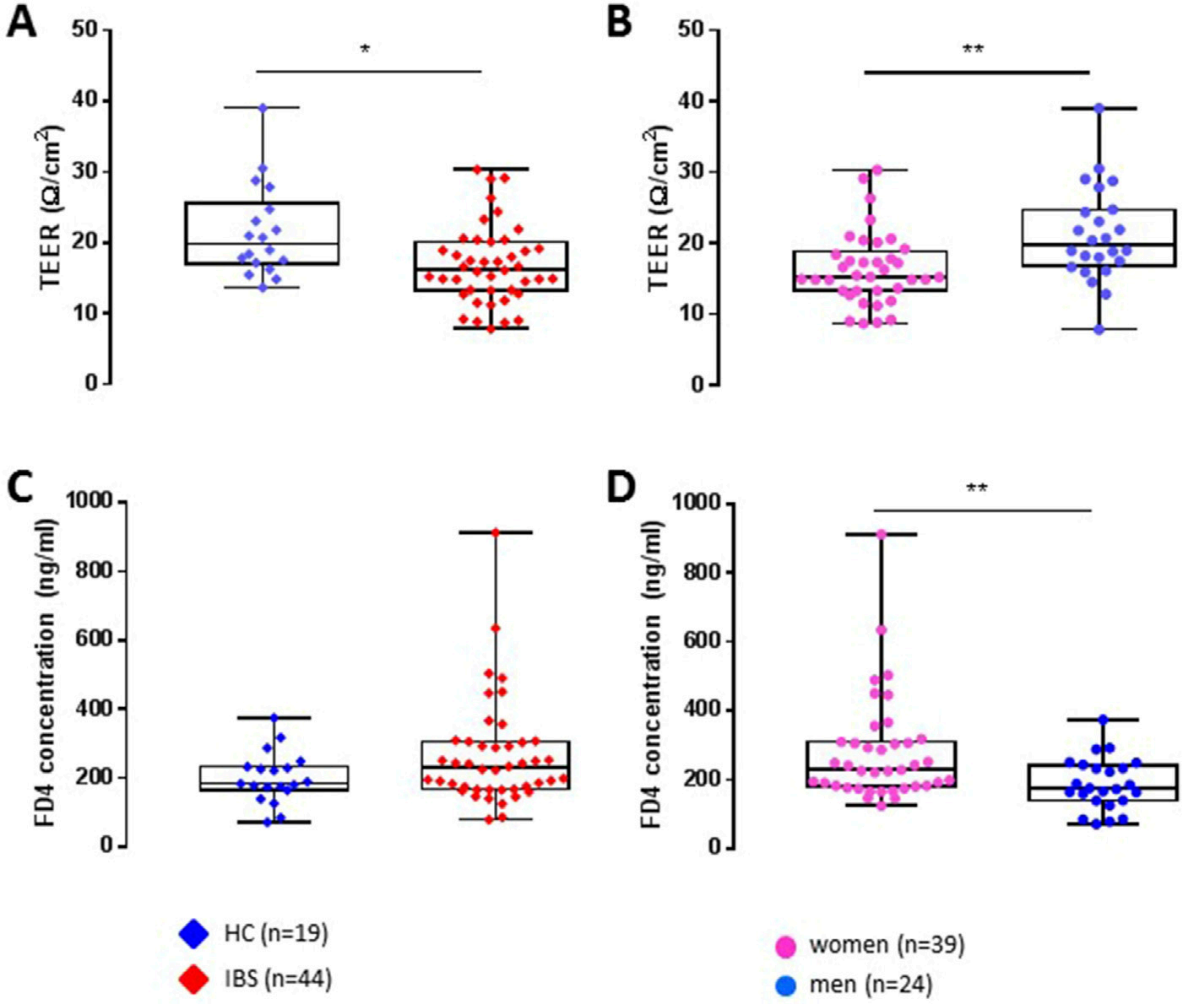
Differences between IBS vs. healthy control groups and sex (women vs. men) on transepithelial electric resistance (TEER) and FD4 permeability (FD4 serosal final concentration) in sigmoid colon biopsies. Taken as a whole group, independently of sex, IBS patients (n = 44) exhibit a decreased TEER **(A)** and no change in FD4 concentration **(C)** compared to HCs (n = 19). When segregated by sex, independently of their disease status, women (n = 39) present a reduced colonic epithelial resistance **(B)** and increased mucosal-to-serosal permeability to FD4 **(D)** compared to men (n = 24). Data are represented as means ± SEM. * FDR<0.05 and >0.01; ** FDR<0.01 and >0.001; *** FDR<0.001. The p-values were calculated within the framework of general linear models, and an FDR <0.05 was considered significant.

Colonic TEER was lower in IBS patients when compared with HCs (Figure 1A) which remained significant after controlling for sex (Table 2). Interestingly, subsequent to the mucosal chamber administration of FD4, biopsies from IBS patients showed similar 2 h cumulative serosal FD4 concentrations (Figure 1C) and FD4 slope (Table 2) vs. HCs, suggesting a trend, but no significant differences in paracellular permeability to FD4 between IBS patients and HCs.

**TABLE 2:** Differences in permeability parameters by diagnostic groups and sexes in sigmoid colon biopsies (IBS vs. HC not covaried for sex).

	Diagnosis								Sex				
Mean (SD)	IBS (n = 44)	HCs (n = 19)	Uncontrolled			Controlled for sex							
			Estimate	p-value	FDR	Estimate	p-value	FDR	Women (n = 39)	Men (n = 24)	Estimate	p-value	FDR
Isc (μA/cm^2^)	80.0 (37.2)	80.3 (24.0)	−0.11	0.58	0.7	−0.14	0.49	0.59	84.3 (38.8)	73.3 (22.6)	0.11	0.415	0.544
TEER (Ω/cm^2^)	16.9 (5.5)	21.5 (6.5)	−0.37	0.01	0.02*	−0.34	0.01	0.02*	16.4 (5.1)	21.1 (6.6)	−0.36	0.002	0.005*
CCh-stimulated Isc (μA/cm^2^)	135.8 (56.1)	133.7 (45.7)	0.01	0.717	0.96	0.01	0.95	0.95	141.3 (55.7)	124.3 (47.4)	0.19	0.295	0.262
[FD4] serosal (ng/mL)	263.9 (152.9)	199.7 (74.6)	0.33	0.122	0.12	0.26	0.15	0.22	280.1 (153.9)	186.7 (75.0)	0.56	0.001	0.005*
FD4.slope (a.u.)	0.7 (0.5)	0.5 (0.2)	0.41	0.155	0.10	0.35	0.08	0.16	0.7 (0.5)	0.5 (0.2)	0.57	0.003	0.005*

a.u., arbitrary unit; NA, not applicable; SD, standard deviation.

Bold values indicate statistically significant differences.

##### 3.2.1.2 Sex differences

Independent of disease status, women exhibited significantly lower TEER (Figure 1B) and higher FD4 serosal concentration (Figure 1D) and FD4 slope compared to men (Table 2), suggesting higher colonic epithelial paracellular permeability to FD4 in women vs. men.

##### 3.2.1.3 Sex differences within HCs or IBS

**FIGURE 2:**
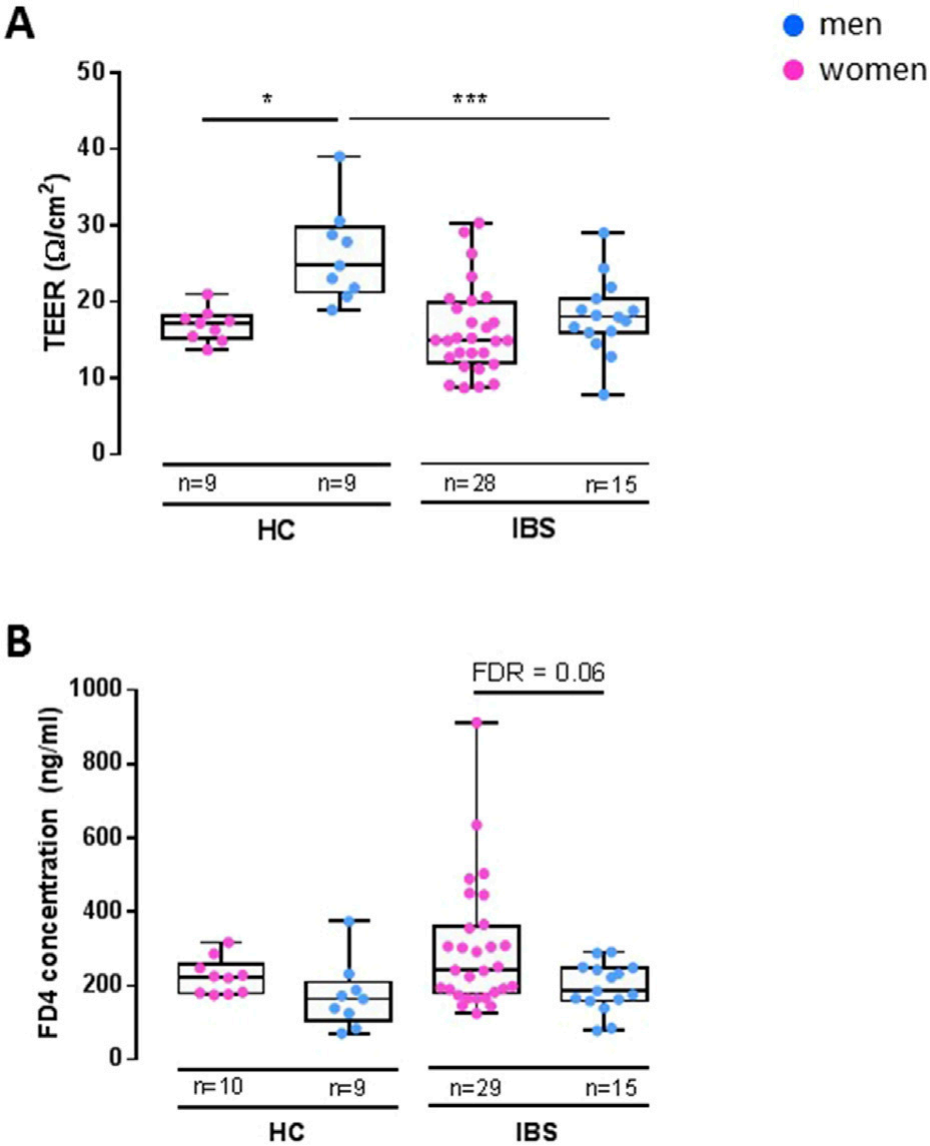
Disease-sex interaction influence on TEER and FD4 permeability in sigmoid colon biopsies. Within HC, women (n = 9) exhibit a lower TEER [**(A)**, FDR<0.05] than men (n = 9), but no change in FD4 permeability. Within IBS, there is no difference in TEER between men (n = 15) and women (n = 28) **(A)**, but women have a higher FD4 permeability than men [**(B)**, FDR = 0.06]. Between IBS and HC, the only difference found is in men with IBS men having lower TEER than HC men [**(A)**, FDR = 9.07 e−3]. Data are represented as means ± SEM. * FDR<0.05 and >0.01; ** FDR<0.01 and >0.001; *** FDR<0.001. The p-values were calculated within the framework of general linear models, and an FDR <0.05 was considered significant.

Within HCs, women had lower TEER (Figure 2A) but no difference in FD4 serosal concentration (Figure 2B) vs. men (Table 3A). Within IBS, TEER was not different between men and women, and FD4 serosal concentration showed a trend to be higher in women vs. men without reaching statistical significance (Figure 2B; Table 3A).

**TABLE 3:** Differences in permeability parameters between diagnostic and sex interaction groups in sigmoid colon biopsies.

	A) Diagnostic group differences within sexes							
	Sex differences within IBS patients (IBS women vs. IBS men)				Sex differences within healthy controls (HC women vs. HC men)			
	Estimate	Effect size	p-value	FDR	Estimate	Effect size	p-value	FDR
Isc (μA/cm^2^)	0.17	0.25	0.44	0.52	0.04	0.06	0.90	0.90
TEER (Ω/cm^2^)	−0.18	−0.44	0.18	0.27	−0.61	−1.43	3.71E-03	0.01*
CCh-stimulated Isc (μA/cm^2^)	0.12	0.21	0.52	0.52	0.36	0.64	0.21	0.25
[FD4] serosal (ng/mL)	0.53	0.84	0.01	**0.06∼**	0.52	0.82	0.08	0.12
FD4.slope (a.u.)	0.48	0.67	0.04	0.12	0.63	0.88	0.06	0.12

	B) Sex differences within diagnostic groups							
	Diagnostic group differences within women (IBS women vs. HC women)				Diagnostic group differences within men (IBS men vs. HC men)			
	Estimate	Effect size	p-value	FDR	Estimate	Effect size	p-value	FDR
Isc (μA/cm^2^)	−0.07	−0.11	0.79	0.79	−0.20	−0.30	0.49	0.58
TEER (Ω/cm^2^)	−0.13	−0.32	0.41	0.62	−0.55	−1.31	3.02E-03	9.07E-03*
CCh-stimulated Isc (μA/cm^2^)	−0.11	−0.19	0.61	0.74	0.13	0.23	0.62	0.62
[FD4] serosal (ng/mL)	0.26	0.42	0.26	0.62	0.25	0.40	0.35	0.53
FD4.slope (a.u.)	0.28	0.39	0.29	0.62	0.43	0.60	0.16	0.32

a.u., arbitrary unit.

Bold values indicate statistically significant differences.

##### 3.2.1.4 Disease differences within men and women

Men with IBS had lower TEER compared to HC men, although IBS and HC women had similar TEER (Figure 2A; Table 3B). No statistical disease differences were detected in FD4 flux within men or women (Figure 2B; Table 3B).

#### 3.2.2 Differences in permeability measurements between IBS bowel habit subtypes

When assessed within all participants, bowel habits had a significant influence on TEER (Table 4), more so in men than in women (Supplementary Figure 2), and was driven by IBS-D compared to HC. Within IBS, bowel habit subgroups did not affect the permeability measures, before and after controlling for sex (Supplementary Table 1).

**TABLE 4:** Influence of bowel habits on permeability parameters in sigmoid colon biopsies in all participants, controlled for sex.

Mean (SD)	IBS-C (n = 11)	IBS-D (n = 16)	IBS-M (n = 11)	HC (n = 19)	F-value	ANOVA p-value	FDR
Isc (μA/cm^2^)	86.6 (40.8)	79.2 (43.2)	78.8 (33.3)	80.3 (24.0)	0.28	0.84	0.84
TEER (Ω/cm^2^)	17.5 (6.9)	15.6 (5.6)	17.9 (4.8)	21.5 (6.5)	4.04	0.01	0.03*
CCh-stimulated Isc (μA/cm^2^)	173.8 (78.8)	131.8 (37.4)	120.1 (37.6)	133.7 (45.7)	2.47	0.07	0.14
[FD4] serosal (ng/mL)	217 (86.8)	272.4 (195.6)	313.1 (46.8)	199.7 (74.6)	2.17	0.10	0.15
FD4.slope (a.u.)	0.6 (0.3)	0.7 (0.6)	0.8 (0.5)	0.5 (0.2)	1.95	0.13	0.16

a.u., arbitrary unit; SD, standard deviation.

Bold values indicate statistically significant differences.

#### 3.2.3 Differences in permeability measures between menstrual cycle phases

Menstrual cycle phase did not correlate with either TEER (Supplementary Figure 1A) or FD4 serosal concentration (Supplementary Figure 1B) in IBS and HC women. Salivary levels of 17β-estradiol and progesterone also did not correlate with these permeability measures (Supplementary Table 2).

#### 3.2.4 Relationships between permeability measures and IBS symptom severity measures

There were significant positive associations between abdominal symptoms unpleasantness 24 h prior to the biopsy sampling and basal Isc and FD4 serosal concentration and slope. However, there were no associations between permeability measurements and abdominal pain severity or IBS-SSS (Table 5).

**TABLE 5:** Association of clinical symptoms with permeability parameters in recto-sigmoid colon biopsies of IBS patients (n = 44), controlled for sex.

	Isc (μA/cm^2^)			TEER (Ω/cm^2^)			CCh-stimulated Isc (μA/cm^2^)			[FD4] serosal (ng/mL)			FD4.slope (a.u.)		
	Std Beta	p-value	FDR	Std Beta	p-value	FDR	Std Beta	p-value	FDR	Std Beta	p-value	FDR	Std Beta	p-value	FDR
Overall Severity (0–20)	−0.153	0.351	0.84	−0.055	0.738	0.88	0.122	0.47	0.8	−0.109	0.513	0.79	0.024	0.887	0.96
Abdominal Pain (0–20)	0.113	0.474	0.84	−0.136	0.395	0.67	0.143	0.376	0.8	0.217	0.179	0.79	0.036	0.825	0.96
Bloating (0–20)	−0.028	0.862	1	0.032	0.842	0.88	−0.03	0.853	0.95	0.091	0.565	0.79	−0.045	0.78	0.96
HAD_Anxiety	0.114	0.474	0.84	−0.026	0.875	0.88	0.159	0.325	0.8	0.065	0.687	0.79	−0.008	0.963	0.96
HAD_Depression	−0.009	0.958	1	−0.132	0.402	0.67	−0.006	0.972	0.97	−0.12	0.451	0.79	0.247	0.119	0.6
Intensity of abdominal symptoms at 24 h (0–20)	0.001	1	1	−0.146	0.361	0.67	−0.063	0.707	0.94	−0.045	0.786	0.79	0.009	0.956	0.96
Unpleasantness of abdominal symptoms at 24 h (0–20)	0.426	0.004	0.04*	0.251	0.108	0.58	0.269	0.087	0.43	0.505	0.001	0.01*	0.479	0.001	0.01*
Intensity of abdominal symptoms immediately after the procedure (0–20)	0.381	0.013	**0.06∼**	0.25	0.116	0.58	0.421	0.006	**0.06∼**	0.068	0.673	0.79	0.021	0.899	0.96
Unpleasantness of abdominal symptoms immediately after the procedure (0–20)	−0.101	0.537	0.84	−0.193	0.244	0.67	−0.117	0.479	0.8	−0.105	0.526	0.79	0.118	0.488	0.96
IBS-SSS	−0.091	0.59	0.84	0.072	0.656	0.88	0.052	0.748	0.94	−0.043	0.791	0.79	−0.014	0.933	0.96

a.u., arbitrary unit.

Bold values indicate statistically significant differences.

### 3.3 Tight junction gene expression

#### 3.3.1 Gene expression differences between IBS diagnostic groups, sexes and IBS × sex interaction groups

When controlled for sex, there were no significant differences in the expression of TJP genes between IBS and HCs (FDR>5%). Compared to men, women exhibited a trend for lower expression of CLDN23 (Table 6). No significant gene expression differences between IBS vs. HC within men or women or sex differences within IBS or HC participants (Table 6).

**TABLE 6:** Influence of disease status (IBS vs. HCs) and sex (women vs. men) on tight junction protein mRNA expression in sigmoid colon biopsies.

	IBS (n = 44) vs. HCs (n = 19) Controlled for sex and seq batch				Women (n = 39) vs. men (n = 24) Controlled for seq batch			
GeneSymbol	Base mean (Log_2_)	Log_2_ fold change (IBS vs. HCs)	p-value	p-adj	Base mean (Log_2_)	Log_2_ fold change (women vs. men)	p-value	p-adj
CGN	2.13	0.19	0.39	0.82	2.13	−0.15	0.13	0.60
CGNL1	−0.19	−0.06	0.79	0.82	−0.19	−0.01	0.97	0.97
CHRM1	−2.14	−0.20	0.12	0.82	−2.14	0.10	0.61	0.76
CHRM3	0.99	0.71	0.01	0.25	0.99	0.52	0.01	**0.07∼**
CLDN1	−1.25	0.31	0.54	0.82	−1.25	0.03	0.81	0.89
CLDN12	0.61	0.01	0.42	0.82	0.61	−0.17	0.38	0.67
CLDN15	0.54	0.06	0.42	0.82	0.54	0.12	0.40	0.67
CLDN2	−2.08	−0.08	0.45	0.82	−2.08	−0.16	0.36	0.67
CLDN23	−0.15	−0.49	0.31	0.82	−0.15	−0.70	0.005	**0.07∼**
CLDN3	3.55	−0.07	0.57	0.82	3.55	−0.18	0.36	0.67
CLDN4	3.75	−0.31	0.49	0.82	3.75	−0.26	0.15	0.60
CLDN7	3.63	0.03	0.71	0.82	3.63	0.12	0.47	0.70
CLDN8	0.60	−0.46	0.54	0.82	0.60	−0.32	0.22	0.67
CMA1	−1.98	−0.08	0.37	0.82	−1.98	0.44	0.03	0.22
CPA3	0.17	−0.25	0.74	0.82	0.17	0.11	0.50	0.70
F11R	2.52	−0.14	0.77	0.82	2.52	−0.21	0.17	0.60
JAM2	−0.60	0.13	0.33	0.82	−0.60	0.03	0.82	0.89
JAM3	0.35	−0.29	0.41	0.82	0.35	0.17	0.28	0.67
MYLK	3.17	0.01	0.65	0.82	3.17	0.05	0.53	0.70
OCLN	−0.88	−0.08	0.73	0.82	−0.88	0.29	0.16	0.60
PLEKHA7	−0.05	0.55	0.08	0.82	−0.05	−0.16	0.35	0.67
TJP1	1.87	0.08	0.60	0.82	1.87	−0.03	0.75	0.89
TJP2	1.79	0.02	0.62	0.82	1.79	−0.02	0.86	0.90
TJP3	1.47	0.07	0.23	0.82	1.47	−0.13	0.52	0.70
TPSAB1	−0.64	−0.20	0.88	0.88	−0.64	0.23	0.36	0.67

Bold values indicate statistically significant differences.

#### 3.3.2 Gene expression differences between IBS bowel habit subtypes

We did not observe significant gene expression differences between bowel habit subtypes controlling for sex (Table 7).

**TABLE 7:** Influence of bowel habits on tight junction protein mRNA expression in sigmoid biopsies between IBS and HCs.

	Uncontrolled for sex			Controlled for sex		
GeneSymbol	F-value	ANOVA p-value	FDR	F-value	ANOVA p-value	FDR
CGN	1.32	0.28	0.63	1.35	0.27	0.61
CGNL1	0.35	0.79	0.89	0.34	0.79	0.89
CHRM1	2.49	0.07	0.46	2.45	0.07	0.46
CHRM3	6.99	0.0004	0.01*	7.53	0.0002	0.01*
CLDN1	1.67	0.18	0.51	1.65	0.19	0.51
CLDN12	0.49	0.69	0.89	0.49	0.69	0.89
CLDN15	0.14	0.93	0.93	0.14	0.93	0.93
CLDN2	0.99	0.40	0.72	1.00	0.40	0.72
CLDN23	2.05	0.12	0.46	2.23	0.09	0.46
CLDN3	0.49	0.69	0.89	0.49	0.69	0.89
CLDN4	1.89	0.14	0.46	1.90	0.14	0.46
CLDN7	1.57	0.21	0.51	1.59	0.20	0.51
CLDN8	1.86	0.15	0.46	1.86	0.15	0.46
CMA1	2.11	0.11	0.46	2.24	0.09	0.46
CPA3	1.01	0.40	0.72	1.01	0.39	0.72
F11R	0.43	0.73	0.89	0.44	0.73	0.89
JAM2	0.82	0.49	0.81	0.81	0.50	0.83
JAM3	1.11	0.35	0.72	1.12	0.35	0.72
MYLK	0.26	0.86	0.89	0.26	0.86	0.89
OCLN	2.07	0.11	0.46	2.07	0.11	0.46
PLEKHA7	2.30	0.09	0.46	2.30	0.09	0.46
TJP1	0.46	0.71	0.89	0.46	0.71	0.89
TJP2	0.27	0.85	0.89	0.27	0.85	0.89
TJP3	0.31	0.82	0.89	0.31	0.82	0.89
TPSAB1	0.33	0.80	0.89	0.33	0.80	0.89

Bold values indicate statistically significant differences.

#### 3.3.3 Gene expression differences between menstrual cycle phases

There was a small increase in expression of mast cell carboxypeptidase 3 (CPA3) in follicular compared to luteal phase (Supplementary Table 3). Due to the small sample sizes, we did not test the menstrual phase-associated gene expression changes between IBS and HCs (n = 21 and n = 7, respectively).

#### 3.3.4 Association of clinical features with mRNA expression

No significant associations were found between IBS symptom severity measures and mRNA expression covaried for sex, after FDR corrections.

### 3.4 Secretory responses: functional assay and gene expression

#### 3.4.1 Differences in basal and stimulated secretory responses between IBS and HCs, sexes and IBS × sex interaction groups

##### 3.4.1.1 IBS vs. HCs

**FIGURE 3:**
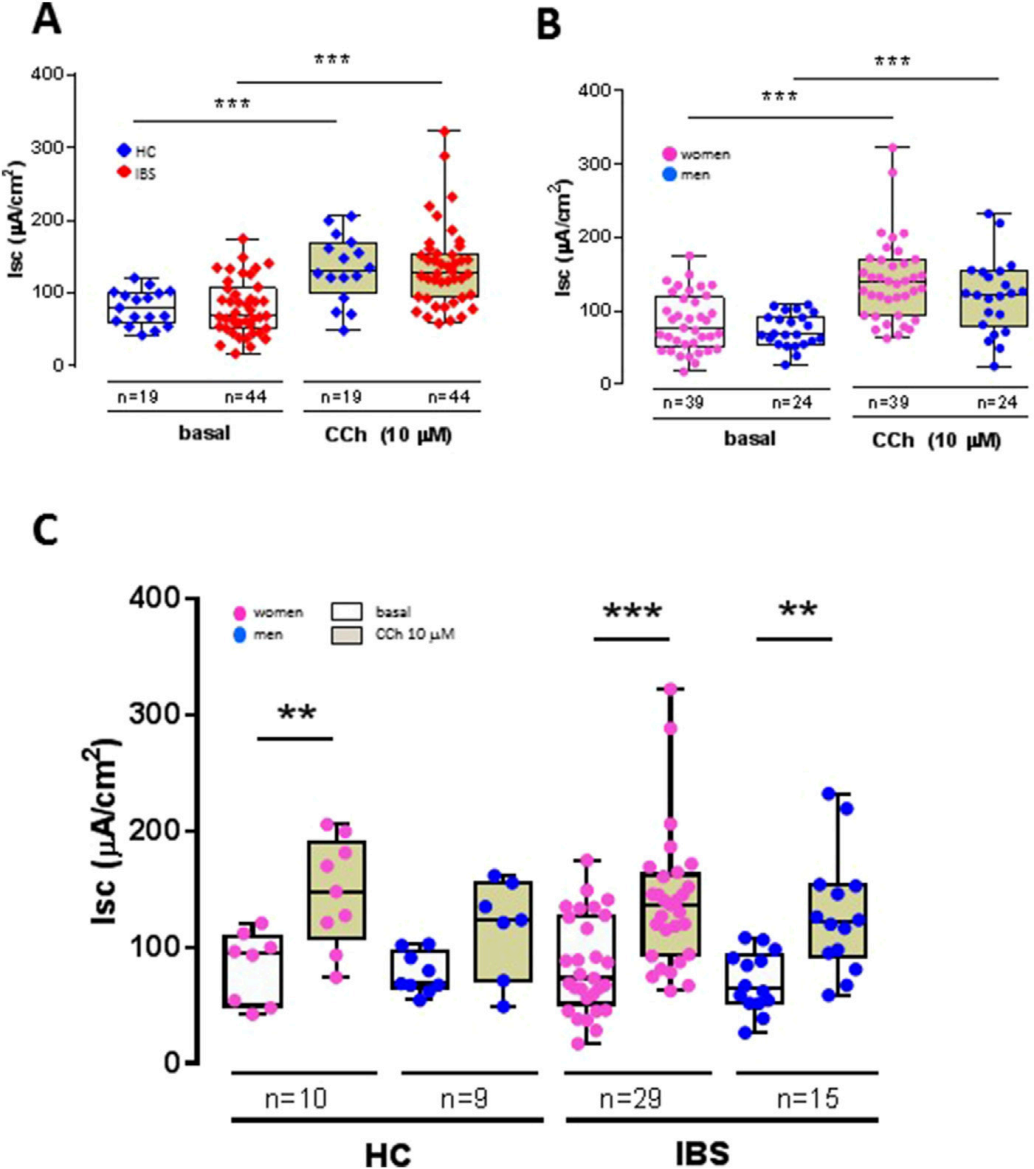
Influence of disease status (IBS vs. HCs), sex (women vs. men) and disease-sex interaction on basal and carbachol (CCh)-stimulated short-circuit current (Isc) in sigmoid colon biopsies. **(A)** Basal active electrogenic anion secretion was characterized by measuring the spontaneous Isc of sigmoid colon biopsies from IBS patients (n = 44) and HCs (n = 19). No differences were found between IBS and HC in both basal and CCh-stimulated Isc, but the Isc in both HCs and IBS showed the expected increased secretory response to CCh, confirming the tissue viability under the experimental conditions. **(B)** Influence of sex on basal Isc and CCh-stimulated Isc in sigmoid colon biopsies from women (n = 39) and men (n = 24). No differences were found between women and men in both basal and CCh-stimulated Isc, but the Isc in both women and men showed an increased secretory response to CCh. **(C)** No differences were noted within and between HC and IBS and women and men. Only CCh significantly increased the Isc compared to basal in each respective group. Data are represented means ± SEM. * FDR<0.05 and >0.01; ** FDR<0.01 and >0.001; *** FDR<0.001. The p-values were calculated using linear mixed-effects models, and an FDR <0.05 was considered significant.

Baseline colon Isc was similar between IBS and HCs (Figure 3A). After maximal stimulation of electrogenic ion transport with carbachol (CCh) (Figure 3A), no significant difference could be detected between IBS and HCs (Table 2). This indicates that the viability of the mucosal samples from the IBS and HCs was not compromised during the course of the *in vitro* experiment and that the disease status does not affect the secretory properties of the sigmoid colon epithelium.

##### 3.4.1.2 Sex differences

Sex had no influence on basal Isc (Figure 3B). Carbachol induced a significant secretory response in both women and men biopsies (Figure 3B) with no difference in magnitude between women and men (Table 3). Compared to men, women exhibited a trend for higher expression of muscarinic acid receptor (Table 5).

##### 3.4.1.3 Sex differences within HCs or IBS

No sex differences were found within HCs or IBS in either basal Isc or CCh-stimulated Isc (Table 3; Figure 3C).

##### 3.4.1.4 Sex differences between HCs or IBS

No differences were found between HC men and IBS men, or within HC women and IBS women (Table 3; Figure 3C).

##### 3.4.1.5 Influence of bowel habits

There was no difference in basal Isc between the IBS bowel habit subgroups. However, IBS-C patients exhibited a trend for higher Isc in response to CCh vs. IBS-D and IBS-M (Table 4). When comparing the gene expression levels between bowel habit subtypes controlling for sex, CHRM3 showed overall differences in expression between bowel habit subtypes. Pairwise comparisons of bowel habit subtype groups controlling for sex suggested possible differences in expression of CHRM3 between IBS-C compared to HCs (p = 0.01, FDR = 0.5).

##### 3.4.1.6 Influence of menstrual cycle phase and sex hormones

There was no correlation between any of the parameters of secretion [basal Isc (Supplementary Figure 1C) or CCh-stimulated Isc (Supplementary Figure 1D)] and menstrual cycle or sex hormones (Supplementary Table 1).

##### 3.4.1.7 Association of clinical features with secretory responses

The intensity of abdominal symptoms immediately following biopsy sampling showed a trend for association with the Isc both basal and CCh-stimulated. No other associations were found between IBS symptoms and secretory parameters (Table 4).

## 4 Discussion

To our knowledge, our study is the first to assess the influence of sex on colonic epithelial permeability and secretion in IBS patients. We found that patients with IBS have increased epithelial permeability compared to HCs, independent of sex. Furthermore, we uncovered a strong influence of sex on human sigmoid colon permeability, whereby women, independent of disease status, exhibit an increased epithelial permeability compared to men. In addition, by analyzing the influence of sex between and within IBS and HCs, we showed that epithelial permeability alterations may play a more prominent role in IBS men than they do in IBS women. Lastly, while secretory responses did not differ between IBS and HC, or between men and women, we established that colonic biopsies from IBS-C patients exhibit a trend for higher secretory response to the parasympathomimetic carbachol and express higher levels of muscarinic receptor 3, suggesting that there are alterations of the cholinergic muscarinic pathways in this subgroup of patients. Our IBS and HC sample size were small however, and larger studies are warranted to confirm these findings.

Increased gut epithelial permeability is an important pathophysiological mechanism underlying symptoms in IBS patients (Hanning et al., 2021). Recently, novel therapeutic interventions aimed at reducing this increase have come forward with promising results for both IBS-C and IBS-D patients (Inczefi et al., 2024; King et al., 2024; Zhou et al., 2019a; Grover et al., 2024). While the prevalence of IBS in women is well established, most of the reports on alterations of gut epithelial permeability have so far not addressed the influence of sex, either due to their small patient size and lack of power to detect sex differences, lack of design to assess sex effects, or due to focus on only one sex. Among the studies that did report SABV, limited sex difference analyses were done (no within or in between group analysis). A few of them indicated sex differences in gut permeability independently of disease, with men exhibiting a trend for higher gastroduodenal permeability (Mujagic et al., 2014) or small intestinal permeability (Marshall et al., 2004) compared to women. In a few other studies, sex did not have any influence (Mujagic et al., 2014; Piche et al., 2009; Keszthelyi et al., 2014). In our patient cohort, similar to earlier studies (Hanning et al., 2021; Witt et al., 2019; Meira de-Faria et al., 2021; Bednarska et al., 2017), we found a lower TEER in sigmoid biopsies (increased epithelial permeability) from IBS patients compared to HCs. We further found that this difference was maintained when controlling for sex suggesting that sex does not play a major role in the epithelial dysfunction observed in IBS patients. Interestingly though, we did not find any increase in the paracellular permeability contrary to prior reports (Witt et al., 2019; Piche et al., 2009). However, in those studies, different paracellular probes were used such as sulfonic acid and ^51^Cr-EDTA which are smaller in size than FD4 and may potentially explain the difference in results.

Importantly, we found that when assessed by sex independent of disease status, women exhibited an increase in *in vitro* sigmoid colon permeability compared to men, shown by both lower TEER and higher FD4 permeability. Previous work using the lactulose/mannitol excretion test in healthy women showed significantly lower *in vivo* colonic permeability than healthy men (Edogawa et al., 2018). In another study, HC women exhibited a trend for higher expression of the tight junction protein (TJP) zonula occludens (ZO)-1 mRNA and protein in colonic biopsies versus healthy men (Lee et al., 2020) suggesting they may have a lower colonic permeability as ZO-1 is a key tight junction controlling epithelial barrier function (Kuo et al., 2021). Still, other *in vivo* permeability assays, which indirectly assessed colonic permeability, did not support sex differences in healthy individuals (Khoshbin et al., 2021; McOmber et al., 2010; Shaikh et al., 2015; Suenaert et al., 2003). Even though there is evidence in preclinical models that the type of assay used to monitor epithelial permeability can affect the direction of sex differences (Volynets et al., 2016), it remains to be determined if this is also the case in humans. Of note, intestinal permeability *in vivo* is determined by both the mucus layer and epithelial mechanisms, whereas epithelial permeability as done in the current study only test the latter. This could also explain some of the differences between studies. Undoubtedly, evaluation of the colonic epithelial function as performed in our study, using biopsies mounted in Ussing chambers, is considered the gold standard of measuring intestinal permeability *in vitro* (Ussing and Zerahn, 1951) and is more likely to directly and reliably inform about changes in epithelial permeability at a specific gut region than other *in vivo* or *ex vivo* assays targeting the whole gut or TJ mRNA expression changes alone, which may not be indicative of actual functional changes.

We explored sex differences in sigmoid colonic epithelial permeability within and between IBS and HCs. HC men had higher TEER than HC women, while there was no difference between IBS men and women. In both IBS and HC, women exhibited higher FD4 permeability compared to men. Disease group comparisons within men and women showed that HC men have higher TEER than IBS men, but similar FD4 permeability, while HC and IBS women have similar TEER and FD4 permeability. Together these data indicate that permeability in men is affected by disease status but not in women. TEER reflects the ionic conductance of the paracellular pathway in the epithelial monolayer, whereas the flux of non-electrolyte tracers indicates the paracellular water flow, as well as the pore size of the tight junctions (Zucco et al., 2005). The fact that TEER is different in IBS vs. HC men, but not FD4 permeability, suggests that IBS affects the colonic paracellular pathway, but not to molecules with a molecular size of 4 kDa. This, however, does not preclude that the paracellular pathway to other smaller molecules or even the transcellular pathway may be affected in IBS men. These findings warrants further study in a larger cohort but nonetheless highlights the importance of evaluating sex differences when investigating permeability alterations in IBS vs. HCs.

Evidence regarding the influence of bowel habits on colon epithelial permeability in IBS patients vs. HCs is very limited (Hanning et al., 2021). In prior studies, *in vitro* colonic biopsy assays showed no differences between IBS subtypes (Witt et al., 2019; Piche et al., 2009; Bednarska et al., 2017). Recently, when assessed within sex according to the disease status, IBS-C women were found to have similar colonic barrier and secretory function to HC women (Peters et al., 2017). In another study, ZO-1 mRNA expression was decreased in colonic biopsies of IBS-D women compared to HC women, likely translating in higher colonic permeability, whereas IBS-D and HC men had similar expression (Lee et al., 2020). In our study, IBS-D, but not IBS-C or IBS-M, had a significant effect on TEER in sigmoid colon biopsies. No significant differences were detected between sex in the different subtypes (Supplementary Table 1). However, our IBS bowel habit subgroup sample sizes were small so larger studies are needed.

One likely factor contributing to sex differences in gut permeability are sex hormones, in particular ovarian hormones, which are well established to vary throughout the menstrual cycle. The influence of the menstrual cycle on GI permeability has been the subject of a few studies (Flood et al., 2022; Shieh et al., 2020; Lambert et al., 2012). As a whole, available *in vivo* data suggest that estradiol levels negatively correlate with gastrointestinal permeability, and this is further supported by *in vitro* studies (Braniste et al., 2009; Looijer-van Langen et al., 2011). Progesterone was also reported to decrease gut permeability (Zhou et al., 2019b). In our study, we did not find any correlation between salivary levels of estradiol and progesterone and the measures of epithelial barrier permeability (TEER, FD4), suggesting that these may be independent from systemic hormonal influence.

Increased gut epithelial permeability in IBS patients has been previously shown to be associated with the severity of IBS symptoms and increased abdominal pain (Camilleri et al., 2012; Piche et al., 2009; Vivinus-Nébot et al., 2014) although not all studies reported such association (Witt et al., 2019). In our cohort, we did not find any association between TEER or FD4 permeability and abdominal pain symptoms.

The passage of nutrient, water and molecules across the epithelial barrier occurs via transepithelial transport, involving the transcellular and paracellular pathways. The paracellular transport, which we monitored in this study, is not very selective and involves the movement of molecules across the epithelial barrier via two pathways: the pore or the leak pathway. Both of these pathways are tight-junction dependent, size-selective, and the pore pathway is charge-selective. Claudin proteins, which form either channels or barriers at the tight junction, define the pore pathway, while the leak pathway permeability, which allows for passage of larger molecules such as lactulose, mannitol and 4 kDa dextran, is regulated among other proteins by occludin, ZO-1 and perijunctional actomyosin (Horowitz et al., 2023). Evidence for alterations in several tight junction proteins expression between IBS and HCs have been reported by numerous investigators (Hanning et al., 2021). In our patient cohort, we did not find any differences in TJP gene expression between IBS and HCs. However, women showed a trend for a lower expression of claudin-23. Claudin 23 has been identified as a barrier forming TJ protein (Günzel and Yu, 2013; Raya-Sandino et al., 2023) and its decrease in expression supports the alterations of TEER observed in women compared to men.

Secretory responses in IBS patients have been little investigated. In earlier reports, when assessed within sex according to the disease status, IBS-C women were found to have similar colonic secretory function than HC women (Peters et al., 2017). In another recent study, the basal Isc, which is the summation of all the ionic currents across the epithelium, was found to not differ between IBS-M and HCs, although a trend to be lower in IBS-M patients was noted (Awad et al., 2023). However, IBS-M patients exhibited a decrease in active chloride secretion indicated by less bumetanide-sensitive rheogenic transport compared to HCs, consistent with the constipation phase seen in these patients (Awad et al., 2023). In contrast, sodium absorption was not affected between IBS-M and HCs (Awad et al., 2023). Similarly, the Isc and ENaC-dependent electrogenic sodium absorption were found to be unaffected in colonic biopsies of patients with post-infection IBS induced by *Campylobacter jejuni* compared to HCs (Omarova et al., 2023). Our study expands and confirms these previous findings and indicates that there is no difference in basal Isc between HCs and IBS patients in a larger group of patients, whether this is analyzed as a group or within each sex. We also show that bowel habits do not have a significant influence on the basal Isc. Interestingly though, in our patient cohort, IBS-C subjects exhibit a trend for higher stimulated muscarinic secretory response to carbachol compared to HCs and exhibit a trend for increased expression of the muscarinic cholinergic receptor 3 (M3R) in the epithelium. M3R is abundantly expressed in epithelial cells in human colon (Harrington et al., 2010) and acts to promote chloride secretion (Hirota and McKay, 2006; O'Malley et al., 1995; Kuwahara et al., 1987). We hypothesize that the increased expression of M3R in the mucosa of IBS-C patients may be occurring as a compensatory response to an impaired cholinergic neurotransmission. Indeed alterations of cholinergic transmission are associated with constipation in elderly patients (Deb et al., 2020), and colonic upregulation of M3R expression has been noted in multiple preclinical models of constipation (Kim et al., 2022; Zhang et al., 2021; Kim et al., 2019). Of note, the changes in M3R in those studies were found in the enteric nerves or the whole tissue, while our results are confined to the epithelium. Whether they do also occur in the enteric nervous system of IBS-C patients is currently unknown.

The present study has several limitations. First, we only assessed permeability changes using TEER and FD4 permeability, which allowed us to evaluate the ionic and paracellular permeability across the membrane. The use of HRP or *E. coli* would have helped us map in a more complete manner the alterations in epithelial barrier function in IBS vs. HC by also assessing the transcellular pathway. We also did not find the alterations of paracellular permeability described by others with the use of smaller sized tracers such as sulfonic acid and ^51^Cr-EDTA, and we cannot exclude that these may have shown differences not detected with FD4. Second, we did not assess tight junction alterations by immunohistochemistry to establish whether RNAseq data have functional relevance at the protein level, and whether some of the tight junction proteins underwent re-localization inside the membrane without changes in expression, which could have given us a more complete view of the sex-differential effects at the colonic epithelial barrier level. Third, our use of the salivary assays to evaluate sex hormones levels may not have been optimal in assessing hormonal fluctuations in our patients (Arslan et al., 2023) and quantification of sex hormones in blood samples would have given us more confidence in the results. Fourth, the subgroup sample sizes are small, and unlike women, our men cohort had a higher representation of IBS-D compared to other subtypes, which could have affected the results, so the presence or absence of significant differences should be interpreted with caution. Lastly, the great majority of studies, including the current one are correlationals and prevent us from making causal inferences.

In conclusion, our study confirms prior reports that IBS patients demonstrate altered sigmoid colonic epithelial function and shows for the first time that these are independent of sex. We also show for the first time that there is a major influence of sex on sigmoid colon epithelial permeability independent of IBS diagnosis, thereby supporting the need to account for sex when studying permeability. Further studies are needed to delineate if intestinal permeability interacts with other factors (e.g., microbiome, immune function) in the pathophysiology of IBS and if these interactions differ by sex.

## Data Availability

The raw data supporting the conclusions of this article will be made available by the authors, without undue reservation.

## Abbreviations

CCh, carbachol; DGBI, Disorders of Gut-Brain Interaction; GI, gastrointestinal; HCs, healthy controls; IBS, irritable bowel syndrome; IBS-C, constipation-predominant IBS; IBS-D, diarrhea-predominant IBS; IBS-M, mixed IBS; IBS-U, unspecified IBS; Isc, short-circuit current; G, conductance; SABV, sex as a biological variable; TEER, transepithelial electrical resistance.
