# Supplementary material for: Sex-dependent alterations of colonic epithelial permeability: relevance to irritable bowel syndrome
Source: Front Physiol. 2025 Feb 21;16:1509935. doi: 10.3389/fphys.2025.1509935 (PMC11885305; doi:10.3389/fphys.2025.1509935)
Supplement: Supplementary file 1 [file DataSheet1.pdf]

SUPPLEMENTARY DATA

Sex-dependent alterations of colonic epithelial permeability: relevance to irritable bowel syndrome

Muriel Larauche[1,2*], Swapna Mahurkar-Joshi[1], Mandy Biraud[1,2], Tiffany Ju[1], Emeran A Mayer[1], Lin Chang[1]

[1] Vatche and Tamar Manoukian Division of Digestive Diseases, David Geffen School of Medicine, UCLA, Los Angeles, CA, USA

[2] VA Greater Los Angeles Healthcare System, Los Angeles, CA, USA

*** Correspondence:**
Muriel Larauche, PhD
mlarauche@mednet.ucla.edu

Supplemental Figure 1. Influence of menstrual cycle phase on TEER and FD4 serosal concentration in IBS and HC women. The menstrual cycle phase of women is color-labeled for luteal, follicular, other (hysterectomy, post-menopausal, menstruating, etonogestrel implant) and unknown (IUD). No significant impact of menstrual cycle can be seen on TEER (**A**) or FD4 permeability (**B**).

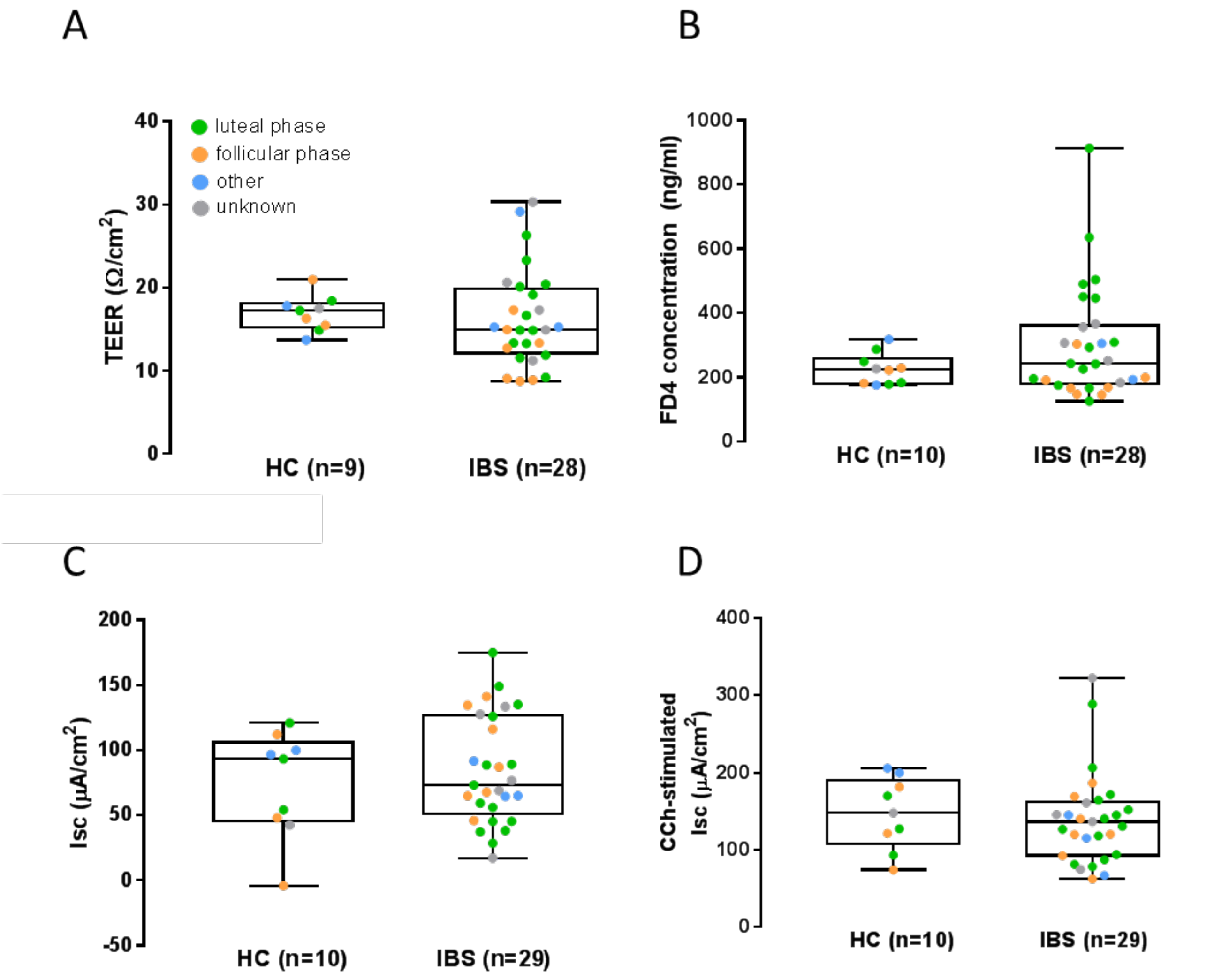

Supplemental Figure 2. Influence of bowel habits on TEER in IBS and HC women (**A**) and men (**B**).

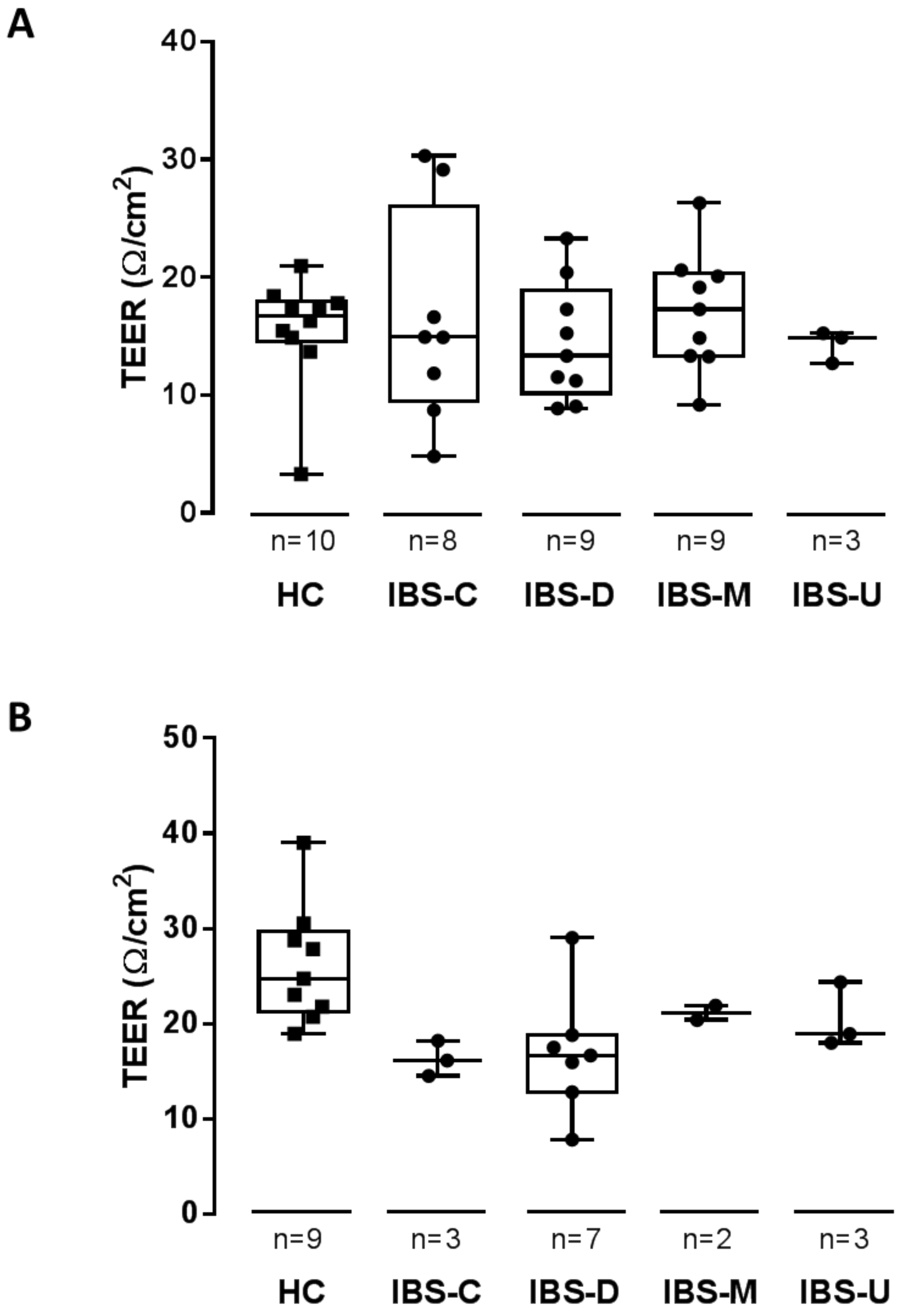

Supplemental TABLE 1: Bowel habit group differences within IBS patients with and without controlling for sex. a.u.: arbitrary unit.

	Not controlled for sex variable			Controlled for sex variable		
	F-value	p-value	FDR	F-value	p-value	FDR
Isc ($\mu A/cm^2$)	0.22	0.80	0.80	0.22	0.81	0.81
TEER (Ω/cm^2)	0.97	0.39	0.58	1.00	0.38	0.57
CCh-stimulated Isc ($\mu A/cm^2$)	3.83	0.03	0.18	3.79	0.03	0.19
[FD4] serosal (ng/ml)	1.09	0.35	0.58	1.24	0.30	0.57
FD4.slope (a.u.)	0.59	0.56	0.67	0.64	0.53	0.64

Supplemental TABLE 2. Correlation between salivary estradiol (E2) and progesterone (Pg) levels and permeability parameters in sigmoid colon biopsies from women independent of disease status.

	**Spearman correlation with salivary E2**				
	**Isc**	**TEER**	**CCh**	**[FD4] serosal**	**FD4 slope**
**r**	-0.05368	0.1035	-0.1357	0.2354	0.1826
**95% confidence interv**	-0.4418 to 0.3514	-0.2986 to 0.4744	-0.506 to 0.2768	-0.1703 to 0.5729	-0.2234 to 0.5346
**P (two-tailed)**	0.7945	0.6075	0.5085	0.2372	0.362
**Number of XY Pairs**	26	27	26	27	27

	**Spearman correlation with salivary Pg**				
	**Isc**	**TEER**	**CCh**	**[FD4] serosal**	**FD4 slope**
**r**	-0.09098	0.08274	-0.06045	-0.02045	-0.03969
**95% confidence interv**	-0.4646 to 0.3101	-0.3176 to 0.458	-0.4402 to 0.3376	-0.1703 to 0.5729	-0.2234 to 0.5346
**P (two-tailed)**	0.6518	0.6816	0.7645	0.9193	0.8442
**Number of XY Pairs**	27	27	27	27	27

Supplemental TABLE 3: Influence of menstrual cycle phase on gene mRNA expression in sigmoid biopsies between IBS-C and HCs.

Gene_Symbol	Contrast	Base_mean	logFC	p-value	FDR
**CGN**	Luteal Phase - Follicular Phase	2.06	-0.03	0.89	0.91
**CGNL1**	Luteal Phase - Follicular Phase	-0.12	-0.29	0.48	0.70
**CHRM1**	Luteal Phase - Follicular Phase	-2.19	-0.29	0.16	0.51
**CHRM3**	Luteal Phase - Follicular Phase	1.26	-0.52	0.01	0.18
**CLDN1**	Luteal Phase - Follicular Phase	-1.01	-0.15	0.76	0.86
**CLDN12**	Luteal Phase - Follicular Phase	0.49	0.21	0.41	0.68
**CLDN15**	Luteal Phase - Follicular Phase	0.52	0.41	0.11	0.38
**CLDN2**	Luteal Phase - Follicular Phase	-2.16	-0.12	0.69	0.86
**CLDN23**	Luteal Phase - Follicular Phase	-0.63	0.33	0.35	0.67
**CLDN3**	Luteal Phase - Follicular Phase	3.36	0.47	0.08	0.35
**CLDN4**	Luteal Phase - Follicular Phase	3.51	0.56	0.05	0.23
**CLDN7**	Luteal Phase - Follicular Phase	3.61	0.31	0.19	0.52
**CLDN8**	Luteal Phase - Follicular Phase	0.31	-0.09	0.72	0.86
**CMA1**	Luteal Phase - Follicular Phase	-1.82	0.21	0.46	0.70
**CPA3**	Luteal Phase - Follicular Phase	0.05	1.03	0.002	**0.05***
**F11R**	Luteal Phase - Follicular Phase	2.29	0.22	0.37	0.67
**JAM2**	Luteal Phase - Follicular Phase	-0.61	0.04	0.91	0.91
**JAM3**	Luteal Phase - Follicular Phase	0.43	0.52	0.04	0.23
**MYLK**	Luteal Phase - Follicular Phase	3.15	0.08	0.57	0.79
**OCLN**	Luteal Phase - Follicular Phase	-0.81	-0.29	0.30	0.67
**PLEKHA7**	Luteal Phase - Follicular Phase	0.005	-0.72	0.04	0.23
**TJP1**	Luteal Phase - Follicular Phase	1.86	0.03	0.82	0.89
**TJP2**	Luteal Phase - Follicular Phase	1.72	0.10	0.33	0.67
**TJP3**	Luteal Phase - Follicular Phase	1.34	0.09	0.70	0.86
**TPSAB1**	Luteal Phase - Follicular Phase	-0.68	0.40	0.38	0.67
